# Satisfaction conditions in anticipatory mechanisms

**DOI:** 10.1007/s10539-015-9481-3

**Published:** 2015-03-06

**Authors:** Marcin Miłkowski

**Affiliations:** Institute of Philosophy and Sociology, Polish Academy of Sciences, ul. Nowy Świat 72, 00-330 Warsaw, Poland

**Keywords:** Representation, Antirepresentationalism, Hard Problem of Content, Satisfaction conditions, Cognitive map, Anticipatory representation

## Abstract

The purpose of this paper is to present a general mechanistic framework for analyzing causal representational claims, and offer a way to distinguish genuinely representational explanations from those that invoke representations for honorific purposes. It is usually agreed that rats are capable of navigation (even in complete darkness, and when immersed in a water maze) because they maintain a cognitive map of their environment. Exactly how and why their neural states give rise to mental representations is a matter of an ongoing debate. I will show that anticipatory mechanisms involved in rats’ evaluation of possible routes give rise to satisfaction conditions of contents, and this is why they are representationally relevant for explaining and predicting rats’ behavior. I argue that a naturalistic account of satisfaction conditions of contents answers the most important objections of antirepresentationalists.

The notion of representation has recently come under fervent attack. The proponents of the dynamic and enactive accounts of cognition suggest that the notion can be entirely eliminated from behavioral and cognitive sciences (Keijzer 2001; Garzon 2008; Chemero 2009; Hutto and Myin 2013). Ramsey (2007) argues that a large number of appeals to representation in cognitive sciences and neuroscience can be explained away in a deflationary manner; in his opinion, simple feature detectors and tracking mechanisms do not warrant genuinely representational talk. He also stresses that a successful theory of representation should meet the challenge of specifying the *representational* role of representational tokens in the cognitive system (this is called the “job description challenge” by Ramsey). A similar challenge, called “A Hard Problem of Content,” has been voiced by Hutto and Myin (2013) who claim that no naturalized semantics to date can account for content with satisfaction conditions. But if both challenges are met, then the charge of the overly liberal use of *representation* is unjustified.

I will answer the job description challenge by solving the Hard Problem of Content. If there is a role for contents with satisfaction conditions in cognitive systems, then this role is *eo ipso* representational. This is why I show how one can naturalistically account for satisfaction conditions of representational contents by offering a template of a representational mechanism. This template may be filled variously, and the particulars are to be decided by empirical evidence. In this paper, only one such filling will be offered.

The structure of the paper is as follows. I first introduce the idea of representational mechanisms to account for current research in ethology, cognitive science, and neuroscience. Then, I present a particular subspecies of representational mechanisms, anticipatory mechanisms, and show how they are sensitive to truth values of their contents. This framework is applied to the example of evaluating future routes in rats as based on current neuroscience. I articulate an intermediate conclusion and argue that anticipation gives rise to truth values of content, and deal with some possible objections. In conclusion, I show how the account of representational mechanisms vindicates a robust notion of representation.

## Representational mechanisms

My strategy for answering anti-representational attacks is to sketch a general mechanistic framework, the purpose of which is to constrain the notion of representation without deciding which features representational mechanisms may have, as this may be discovered only empirically. The mechanistic framework is now one of the most successful accounts of explanation in special sciences, and has been used to analyze causal explanatory strategies in special sciences. For a recent review, see Illari and Williamson (2011). While definitions of mechanisms offered by various authors accentuate different aspects, the main idea can be summarized as follows: mechanisms are complex structures, involving organized components and interacting processes or activities that contribute jointly to a capacity of the structure. Mechanistic explanation is a species of causal explanation, and interactions of components are framed in causal terms.

There are advantages to using this framework. First, the mechanistic explanation requires that we specify the capacity or capacities of the mechanism (the explanandum phenomenon), which is then explained causally. This can serve to naturalize representation. Second, mechanistic explanation focuses on the organization of the system, and it requires that the exact role of a representation be specified. Representations cannot float freely without being part of a complex system.

My first step is therefore to describe the phenomenon to be explained, or the capacities of representational mechanisms. I will specify these capacities in representational terms and sketch how they are interrelated, without going deeper into their possible causal bases. The second step is to show a particular case study of an anticipatory representational mechanism that actually explains the representational phenomena. In other words, the burden of naturalizing of representational phenomena is shifted to particular causal explanations. It is obviously not enough to define representational capacities to naturalize them; the causal bases have to be given as well. But these causal bases are discoverable only empirically, and they cannot be reliably derived from one’s armchair. Moreover, representational capacities are specified here abstractly enough to be realized in a variety of different mechanisms with different causal structures. The only things that can be specified in a philosophical analysis are the requirements that such causal explanations should satisfy. This much—and only this much—is provided in this section of the paper.

While representational mechanisms may be used to explain larger mechanisms, I focus on them as explananda. Depending on how the explanatory problem is posed, the capacity of a representational mechanism may be framed in various ways. What is common is that the representational mechanism has the capacity to make some information available for the cognitive system (Miłkowski 2013). The information in this case becomes *semantic* as far as it modifies the readiness of the system to act one way or another. More precisely, the conditional probabilities of actions of the system are modified appropriately given the information (MacKay 1969).

By framing the capacity of the representational mechanism as modification of the readiness to act, based on the information available to the cognitive system, this framework is committed to two theses. First, representation is essentially action-oriented, though this orientation does not mean that all representations directly activate effectors of the system, or that representation simply controls the motor activity of the system. There might be content that is not exploited in action; what is altered is just the *readiness* to act. The notion of action is to be understood liberally to include cognition. Second, the account makes use of the notion of information. Though there are various mathematical measures of information, they should not be confused with the notion of information, as MacKay (1969) stressed. All we need for our purposes here is that there is a physical medium with at least two degrees of freedom (or two levels of logical depth) that make a difference to the behavior of the system; in other words, the system reacts differently to at least two distinct states of the physical medium. The notion of information introduced informally here is equivalent to structural information.^1^

Typical explanatory texts cite other important capacities of representational mechanisms. The notion of representation is introduced to talk about targets (or referents, or extension) of the representation, and to talk about the characteristics of the targets (or intension). In addition, the information cannot simply *sit* in the mechanism; the system has to care about it somehow. Thus, there are at least three other essential capacities of the mechanism in question:

- Referring to the target (if any) of the representation;
- Identifying the characteristics of the target;
- Evaluating the epistemic value of information about the target.

While the first two capacities bear close resemblance to traditional notions of extension and intension, the third is supposed to link the mechanism with the work of the agent or system.

One might object that including the characteristics of the target violates the principle of parsimony. For example, proponents of the causal theory of reference dispose of the remnants of the idea of intension altogether (Fodor 1992). But referential opacity, which is arguably a mark of genuine representation (Dennett 1969), is easily explained by appeal to the characteristics of the target. Without at least minimal access to the characteristics, intensionality is hard to pin down.^2^ By including the characteristics of the target as a feature of the representational mechanisms, I do not suggest that it is impossible for (almost) purely extensional representations to exist. The characteristics might be very minimal indeed, such as bearing a particular label or being in a *mental file* (Récanati 2012). The account of representational mechanisms is ecumenical: whether a given system uses a richly-structured medium or not is a matter for empirical investigation, and not for armchair conceptual analysis.

The third capacity of the representational mechanism, namely evaluating the epistemic value of information about the target, may seem misplaced, and not directly related to representing at all. Granted, sometimes it may be beneficial to abstract away from such factors; for example, formal grammars are usually not related to any epistemic values. Nevertheless, in many psychological and behavioral theories, a complete story about representing is linked to a story about the adaptive value of the representation for the organism in question.

These three representational capacities are interlinked. The semantic information that modifies the readiness for action of the cognitive system, which may be in the form of characteristics of the target or simply indicate the target, is what is epistemically evaluable. To evaluate the information already present in the system, the mechanism needs to be able to compare two sets of characteristics of the target. Epistemic evaluation requires more than negative feedback in the information-processing mechanism: this feedback simply modifies the system’s input value. What is required instead is that the error is detected by the system. The idea that system-detectable error gives rise to genuine representationality is by no means new. For an extended argument, see Bickhard (1993).

What are the mechanisms that can display representational capacities? The neomechanistic answer to this question is: those whose parts and operations contribute causally to certain patterns of their functioning which can be seen as specifying or modifying their characteristics of targets, which can be predicted and explained as referring to targets, and which have certain information-related operations. In other words, all usual pragmatics of mechanistic explanation apply here; the explanation should offer new predictions, be general, the entities should be causally relevant, and the causal model of the mechanism has to include all the parts and operations of a representational mechanism. This may sound somewhat vague but the thought is straightforward: if there is no explanatory gain in positing representational capacities in a system, the mechanism in question is not to be explained representationally. That is to say, if there is a simpler causal explanation of a mechanism, and it has the same predictive power related to different interventions in the mechanism, a more complex explanation has to be rejected as spurious.

A chair standing on a floor is not explained correctly as the mechanism that displays a capacity to think that it is located where it should be. Even if one could produce such a spurious explanation and stipulate that the chair as a whole is a bearer of information that characterizes its best location, there are no parts of the chair whose function is to specify that chair or its optimal placing (for example, by being similar to the chair). The chair also does not detect any error if it is moved, and there are no multiple levels of organization of the chair where information vehicles and error information play relevant causal roles. Finally, all such “explanations” are spurious and trivial as they cannot supply any *new* true predictions.

Let’s take a slightly more complex system: a mouse trap. It is not usefully explained as desiring to catch mice even though intentional stance descriptions and predictions of the trap’s operation may be correct though trivial. This is because there are no information bearers of the alleged desire that would be changed appropriately if the mouse trap catches my finger rather than a mouse. For this reason, there is no capacity to evaluate the desire allegedly satisfied in a trap by catching mice. So even if a mouse trap could be used as a (fallible) mouse detector, its functioning does not include any operations on information bearers which could be understood as correcting or signaling errors.

But what about simple negative feedback systems such as the notorious Watt governor? I submit that these are not representational mechanisms, either. The Watt governor is a mechanical device for stabilizing the speed of the steam engine and its work is usually explained in terms of control theory. It is used as a metaphor for the dynamical account of cognition (Van Gelder 1995). The governor is formed by two heavy balls on a frame that is driven by the engine. The centrifugal force makes them go up or down, which closes and opens the steam valve. So, there is negative feedback between the speed as detected by the centrifugal governor and the engine. Yet even if one analyzes the Watt governor in information-processing terms, as Bechtel (1998) or Nielsen (2010) do, the structure of the system does not contain any error-processing routines. Instead of evaluating and modifying its previous *output information* (the “signal” to open or close the valve), it simply changes its operation by having different input (balls move up or down). Thus, the Watt governor does not detect any error in the information: although in the eyes of the beholder, it might have misrepresented the state of the steam engine that it controls; the error is not system-detectable. Hence, the present account is not as liberal as the previous mechanistic analyses of the Watt governor (Bechtel 1998). There is no special representational role to be played by the states of the governor.

System-detectability of error may be realized in various ways. For example, the system may have multiple independent sources of information (Dretske 1986). The system might also use the characteristics of future action of an organism to control the action A of the system and to check whether A is successful; if the success of A is presupposed by another action B, the lack of success falsifies the information contained in the presupposition related to these characteristics (Bickhard 1993). The third solution is to use previous input information to predict the future input state and then to compare with that state. Although the two latter solutions are somewhat similar to negative feedback, they are different in that they imply the processing of information on multiple levels. This is typical, for example, for contemporary predictive processing frameworks (Hohwy 2013; Clark 2013), in which a model of the future state of the system is built, and then the model is compared with that state, when it occurs; from a technical point of view, the model becomes another source of information, though not statistically independent. A complete description of the representational mechanism needs to detail the structure of the evaluation subsystem by specifying the exact way that the mechanism detects vehicular discrepancy between two pieces of input information or between the model and the input information.

A view close to the one presently defended has been advocated by Burge (2010). Burge claims that a necessary condition for the substantial use of the notion of representation is that there is a non-trivial use of veridicality conditions in the theory that ascribes the representation to a creature. Burge answers Ramsey’s challenge by placing veridicality as a necessary condition of application of the notion of representation. The paradigmatic case of non-trivial appeal to veridicality for Burge is perceptual constancy, for example, size constancy. The retinal image of an object varies with distance but the perceived size of the object remains the same. However, perceptual constancies may be present in virtue of different mechanisms operating on various time-scales. Some might involve simply tracking rates of change in the stimulus, and others complex integration of sensory inputs. Thus, in some cases there might be error-checking (and veridicality) involved, while in others the appeal to representation may be superfluous.

Burge stresses that veridicality is crucial, whereas error-based accounts of representation consider the lack of veridicality as the paradigmatic case of error. Even if error detection is implemented as a discrepancy check in the neural system, not all discrepancy checks count as representational error detection. Consider a robot that scans for an accidental change in raw data by using a standard error-detecting code. Such raw data is not necessarily the representation of the robot unless the data is used in such a way as to justify the claim that it refers to or describes something. Mere error-checking is not enough; and the stress on veridicality is correct insofar as description of the functioning of the representational mechanism needs to invoke the target being represented (or misrepresented). If the mechanistically correct, complete explanation of the robot’s functioning mentions no target, and no characteristics of the target, there is no need to posit representation.

The framework of representational mechanisms specifies their capacities and describes conditions that the various mechanisms must fulfill in order to qualify as representational. These conditions are both necessary and sufficient, and though highly abstract, they guarantee not only that the mechanisms store and process information that modifies their readiness to act, but also that they are, at least in some cases, able to refer to targets, identify them, and evaluate the value of the information stored. This way, representational mechanisms are able to detect that they are in error (via evaluation of the epistemic value) and, at least in some cases, are prone to misidentification of targets because of referential opacity. Both aspects, namely system-detectable error (highlighted by Bickhard) and referential opacity (emphasized by Dennett), have been discussed as possible bases for causal relevance of content as content. We will see this in more detail in the example of prospective planning of routes in rats, introduced in Anticipating future paths in the maze section of this paper. It will also causally explain how satisfaction conditions of content arise.

## Anticipatory mechanisms

Anticipatory representational mechanisms not only represent but do so in an anticipatory manner; they anticipate the future characteristics of the represented target. Such capacities are widely posited in current cognitive science and neuroscience as related to conditioning (Schultz and Dickinson 2000; Lauwereyns 2011) and forward models (Pickering and Clark 2014), but the conception has its roots in Helmholtz’s idea of unconscious inferences as inherent in active movements of the eye: a motor signal from the central nervous system is sent both to the motor system and, as a copy, to an internal forward model (Meulders 2010).

In this paper, I distinguish *anticipation* from *prediction*. The latter term may be used to refer to a process of inferring current events; for example, in the predictive coding framework, the brain is usually tasked with predicting current sensations, given their causes—only in generalized predictive coding does the task potentially cover both current and future sensations (Friston et al. 2011). In what follows, I assume that only predicting future sensations is anticipatory. Therefore, the current account does not imply the predictive brain theory of (Hohwy 2013; Clark 2013), and is merely consistent with the latter. But some of the brain’s predictions may be anticipatory. As there is a significant role for error correction in the predictive brain theory, mechanisms posited by this theory to deal with future sensations will be anticipatory representational mechanisms.

To posit an anticipatory representational mechanism in a cognitive system is to reject two claims. First, the current account rejects anti-representationalism. These anticipatory mechanisms meet Ramsey’s challenge; by appeal to them, one can show the way in which representation is both causally relevant and useful for cognitive systems. They also solve the Hard Problem of Content. Specifically, the anticipatory representations have truth values and can be falsified (or confirmed) by the cognitive system on its own. Second, it rejects the simplistic idea that representing is nothing over and above having information about past and present sensory stimuli. But what is the positive claim?

Let me introduce the idea of a modeling relationship as presented by Rosen (1991, 2012), which can be used to shed some light on anticipation. His theory is framed in terms of category theory, but the idea is straightforward. The natural system is modeled by a formal system if and only if the causal process in the natural system is congruent with three elements: (a) measurement of a value of the natural system; (b) inference on that value; and (c) the inferred value in terms of some physical quantity. This means that there is a mapping (at least a homomorphism) between two entities: (1) measurement values encoded by the formal system, inferences on values, and inferred values, decoded in terms of physical quantities, and (2) the natural system.^3^

Having defined the modeling relationship, we can now define the anticipatory system as one “containing a predictive model of itself and/or of its environment, which allows it to change state at an instant in accord with the model’s predictions pertaining to a later instant” (Rosen 2012, 312). Based on values measured in the cognitive system itself or in its environment, the model is fed with these values and used to predict their later states. Without the model there would not be any changes of state in the anticipatory system (nor would it be an anticipatory system in Rosen’s sense).

One important distinction between two kinds of anticipatory systems was introduced by Dubois (2003). *Weak anticipation* (or *exo*-*anticipation*) uses externally-produced data to internally model future states of the environment, while *strong anticipation* (or *endo*-*anticipation*) uses internally-produced data to model future internal states [cf. also (Collier 2008)]. Some authors argue that strong anticipation is not necessarily related to internal models and point to the phenomenon of anticipating synchronization (Stepp and Turvey 2010). In this case, however, strong anticipation might be reducible to weak anticipation, at least explanatorily. Especially in the case of coupled and synchronized systems, one might be tempted to eliminate the notion of representation altogether. However, in anticipatory representational mechanisms, the organization of the mechanism requires more than anticipating synchronization.

The anticipatory representational system has the capacity to derive its own future state, and that future state is evaluated epistemically, for example by comparing whether there is a vehicular discrepancy between expected sensory information (the future state) and actual sensory input. The discrepancy is typically used to correct the error for future derivations of states. Any anticipatory system that has these error correction mechanisms is already a representational mechanism in the sense used here. The anticipation is generated to be evaluated later. Hence, it has truth-value and can be falsified by the system. Mere structural information cannot be falsified but a prediction about the future state of the system can. However, if there is no error correction, there need not be any truth evaluation involved. For example, anticipating synchronization in strongly-coupled dynamical systems does not have any role for error correction, and it therefore does not need to be representational at all.

Hutto (2013) argues that action-oriented accounts of representation cannot offer a viable notion of causally relevant content because “the contents of representations do not make a causal difference, only formal or vehicular properties do;” when one assumes computationalism, then the only causally relevant factors are forms of symbols in computers, irrelevant of their semantics. Though it has been recently argued that computation may be essentially content-involving (Rescorla 2012), I will not pursue this line of argument here. Let’s assume that only formal or vehicular properties make causal difference in computers. The work of my computer would be explained by referring to software and hardware components involved; in particular, one is able to explain how the words I had typed appeared on the screen. This seems right; my computer does not know the truth value of my statements.

But the existence of a computational explanation does not exclude the possibility that that there is a deeper cause of the words on the screen. Obviously, without learning English, I wouldn’t be able to type these meaningful sentences. There is another larger causal explanation, the one related to the content of my statements that I wanted to include in my paper. The computational explanation is just a part of that complete representational explanation (Miłkowski 2013), which also includes my mental representation and contents.

The complete representational explanation has a different phenomenon to explain: not just the appearance of pixels on the screen but also the appearance of words *qua* meaningful bits on the screen. Only some pixel configurations correspond to meaningful statements, so while formal computational explanations explain why a particular configuration appeared, they do not explain why it was one that corresponds to a meaningful statement, the one that has a truth-value. Hutto conflates a proximate cause of the appearance of words with a distal one, and does not see that there is a further fact to be explained. But the proximate cause does not explain why only some configurations of letters appear on my screen, given the fact that the keyboard is able to send various signals to my computer.

Even if one grants that computational mechanisms are sensitive only to vehicular properties, it does not follow that I am not sensitive to satisfaction conditions of my representations. My computational mechanisms make it possible to compare expectations with current states of affairs, and while the comparison may be purely computational, it is embedded in a larger process which makes it semantic at the same time; that process controls my behavior and definitely involves truth conditions. Otherwise, why should I correct myself when I make a typo? Why shouldn’t I just press a random sequence of keys? Hutto has no credible answer to these questions, and his passing the buck to social practices is a non-starter without an account of social emergence of truth values.

## Anticipating future paths in the maze

Cognitive maps in rats have long been a topic of debate in psychology (Tolman 1948). For a theory of mental representation, they are a specially interesting case. They are structured, even compositional, but not reducible to language-like symbol media (Rescorla 2009); they constitute an instance of what Cummins (1996) calls *S*-*Representation* without being simply picture-like representations. But for mechanism, a complete explanation requires putting cognitive maps in a broader context of the brain architecture. In particular, it is extremely important to link maps with actions and evaluation mechanisms linked with reward subsystems in the brain.

At first, cognitive maps seemed to be necessary for explaining the navigational capacities of rats, but later the role of cognitive maps was questioned; even if contemporary neuroscience vindicates the existence of cognitive maps by locating them in the hippocampus (O’Keefe and Nadel 1978; Redish 1999; Derdikman and Moser 2010), it is still not universally accepted that rats exploit them in navigation. Tolman wasn’t overly clear when he introduced the term ‘cognitive map’, and gave no definition in his seminal paper. One may enumerate at least three meanings that commonly occur in the literature: (1) the *trivial* meaning, in which a cognitive map is any mechanism involved in spatial navigation, (2) the *loose* meaning, in which the map is simply any representation that models geometric aspects of the environment, and (3) the *strict* meaning, in which cognitive maps have a format typical of maps, so that they not only represent geometric aspects but do so in a geometric manner (Rescorla 2009, 381). Cognitive maps in the trivial sense are always present in all spatially navigating animals. The debate about the existence of cognitive maps focuses on the loose and strict renderings of the notion.

The reason given for skepticism that rats have *cognitive* maps is that even in circumstances in which they would be useful, rats are unable to reach their goals (Whishaw 1991; Benhamou 1996). They prefer simple visual cues, so there seems no reason to assume multimodal cognitive mapping in rats; their behavior would be fully explainable in terms of tracking environmental cues, just like tropisms or taxis are used in simpler organisms. Invertebrates navigate successfully without multimodal integration (Cruse and Wehner 2011).

This is why proponents of radical enactive cognition (REC), such as Hutto and Myin (2013), can claim that a rat’s mind is, to use their phrase, a basic mind without content. Though Hutto and Myin do not define the notion of the ‘basic mind’, it seems to refer to animal minds and to human minds not engaged in any linguistic activity. Proponents of REC would admit that there are structures in a rat’s brain that carry information about the environment, but they claim that information does not constitute truth-evaluable content. Hence, the S-representation account of cognitive maps (Cummins and Roth 2012) cannot be used to argue against REC. Also, even if it is undeniable that the notion of representation has been used heuristically in biological research on rat navigation (Bechtel 2014), it does not mean that there is a viable naturalist account of truth-evaluable content as embodied in the brain. What is missing is a substantial account of satisfaction conditions of various representations involved in rat navigation.

Rat navigation is quite complex. Behavioral experiments confirm that, at least in some cases, rats are able to return to their starting position, even if they were exploring the environment in complete darkness, devoid of smell (in a water platform or a water maze), and without whiskers to orient towards walls, simply by using their motor signals and vestibular system (Cheung et al. 2012). One problem with confirming a hypothesis about cognitive maps is that experiments need to be conducted in darkness, and one of the kind of neural cells responsible for navigation—head direction cells—becomes unstable after 3 min in darkness, while place and grid cells (other components of the neural system of navigation) fire stably for half an hour longer. Cheung et al. (2012) have shown that using maps beyond 3 min is theoretically implausible and landmarks alone cannot suffice for a stable positional representation. Therefore, a complex organization involving both maps and cue-tracking emerges.

Navigation in rats uses various kinds of information, and the hippocampus in rodents has the function of integrating various sources of information (Lisman and Redish 2009). There are hypotheses (Conklin and Eliasmith 2005) that this integration involves using sensory information for error correction as rats obviously make mistakes in their navigation. For example, misidentification of location is possible as long as the structure of the environment matches the structure encoded by place cells, and even if the mistake is undetected by the rat, it is explainable by assuming that the rat misrepresented the current or future locations (Ferbinteanu and Shapiro 2003). Referential opacity is therefore possible for cognitive maps: the structure of the environment as represented by the cognitive map may match two targets, but only one would be the proper one considering the existence of goals of the animal. Because the animal makes mistakes in orienting towards its goals in such a geometrically similar environment, we must explain its mistakes as being caused by misrepresentation.

While the hippocampus plays multiple functions, there is substantial empirical evidence that landmark location is retrieved in an anticipatory fashion.^4^ The hippocampus is known to be related to memory function, and one of the fundamental features of memory is control of current behavior. It also directs behavior to receive reward and avoid negative consequences. This in turn requires using memory to predict the outcomes of more complex actions. Currently, at least two predictive mechanisms related to evaluating future paths towards valuable goals in rats are known: one related to temporal encoding, where cells encoding future locations are activated along with current ones on place fields, and another with sharp-wave-ripple effects. I will discuss each.

Experiments show that many spikes fired by place cells actually represent a position ahead of the rat (Johnson and Redish 2007; Lisman and Redish 2009).^5^ These spikes are called *sweeps*. For example, if the rat place cells encode a sequence of places A, B,…, G, then when it is located on A, the G cell will also fire. Sweeps do not occur when the animal is on a running wheel. Hence, there is a specific way of modeling the future location in place cells (using phase precession that does not occur normally, so the rat can distinguish between anticipating and actually being located at the landmark). These anticipations are only about locations less than a meter from the current position of the rat where the animal will usually arrive in a few seconds.

Mere evidence for anticipatory encoding of location does not suffice to establish that the path integration mechanism is actually representational. As I claimed above, an evaluation subsystem is also needed. This is exactly what is hypothesized in the recent research on rats: while rats stop at difficult places in the maze and the hippocampus engages in sweeps related to future paths in the maze, the ventral striatum is the evaluation subsystem (van der Meer and Redish 2010). In terms of computational neuroscience, these brain areas form a so-called actor–critic architecture; one executes and plans actions, and another sends a signal about the correctness of actions (basically, a prediction error). While actor–critic algorithms are prevalent in the literature on reinforcement learning, not all reinforcement learning algorithms require models. Shea (2014) defends a claim that even basic temporal difference algorithms are substantially representational. This would mean that REC cannot possibly be right about any organism capable of reinforcement learning. Enactive basic minds would be indeed very basic, and there would be no meaningful application of REC to higher animals, not to mention humans. However, Shea’s position is stronger than the one defended here; he claims that reward signals are meta-representational, whereas in the current account the error information needs not be representational (which would require an infinite regress of evaluation subsystems for reward signals to make them representational). The reward signal is taken literally as a signal of vehicular discrepancy between predicted and actual reward. It is only the prediction that is representational in virtue of being evaluated.^6^

Still, there are kinds of temporal difference learning that are model-free.^7^ The notion of the *model* as applied to reinforcement learning is understood as an internal map of events and stimuli from the external world. As Dayan and Berridge explain:

That internal model supports prospective assessment of the consequences of taking particular actions. By contrast, model-free strategies have no model of outside events; instead, learning takes place merely by caching information about the utilities of outcomes encountered on past interactions with the environment. This generates direct rules for how to behave, or propensities for performing particular actions, on the basis of predictions of the long-run values of actions. Model-free values can be described as being free-floating, since they can become detached from any specific outcome. (Dayan and Berridge 2014: 473)

A stronger case for representationalism would be model-based reinforcement learning, with rich content that has satisfaction conditions. Model-free reinforcement learning might rely merely on cached values and does not have access to information about consequences of actions, which is required for planning of complex tasks. It is inflexible; “because such cached action values are based only on actual rewards received in the past, they cannot support latent learning, are not available in novel situations, and are only reliable if the world does not change too rapidly relative to the speed of learning” (van der Meer and Redish 2010). Including models makes the organism much more flexible.

Rats are indeed flexible in planning their routes. The particular architecture posited to explain sweeps and the work of the ventral striatum, or *dynamic evaluation lookahead*, requires forward models. Dynamic evaluation lookahead is defined as “evaluation of a future outcome that takes the agent’s current motivational state into account” (van der Meer and Redish 2010). It is a two-step process that requires prediction, then evaluation, of the outcome, mapping the prediction onto a value usable for decision making. A forward model allows the animal to predict the outcomes of different available actions; in contrast to simple Pavlovian reinforcement learning, where the learned association is activated. There is evidence that ventral striatum neurons respond to actual reward receipt and to cues that predict it; and this dual encoding is required for a component to play a critic role in the actor–critic architecture. Yet there is no direct evidence for some parts of the proposed explanation of the rat’s behavior. For example, little is known of the mechanism by which expectancies become linked to particular actions. The actor part, the place cells, is better known, as the research on the encoding began in the 1970s with the seminal work of O’Keefe and Nadel (1978). There is little doubt that the place cells encode both structure of the environment and the routes to be taken towards valuable goals. The properties of the place cells indicate that they carry structural information about the environment and that information is used for control or to modify the rat’s readiness to act. The structure can be evaluated, and ventral striatum achieves this.

The second mechanism involved in representing future paths is related to finding trajectories to a goal. The rat’s hippocampus generates brief sequences encoding spatial trajectories strongly biased to progress from the subject’s current location to a known goal location. Pfeiffer and Foster (2013) were able to find *direct* evidence for the existence of future-focused navigational activity of place cells in a realistic two-dimensional environment. They have elegantly shown that it is related to sharp-wave-ripple (SWR) events; SWRs are irregular bursts of brief (100–200 ms) large-amplitude and high-frequency (140–200 Hz) neuronal activity in the hippocampus. However, it is unclear how this mechanism is related to the first one, and whether or not it uses the same evaluation subsystem. The researchers are investigating the functional relevance of trajectory events in guiding behavior, particularly in terms of how the hippocampus interfaces with brain regions involved in reward learning and reward-based decision making.

Let me summarize. I have argued that current neuroscience has evidence for anticipatory representational mechanisms involved in rat spatial orientation. Rats are able to detect errors in their representation, which is related with reward-based decision making and reinforcement learning, and this fulfills one of the criteria for representational systems. The spiking activity of place cells (both firing rates and phase precession) gives rise to a representation that has satisfaction conditions. The properties of firing are vehicular properties, and vehicular properties do not have satisfaction conditions. To conflate content/vehicle distinction is to commit a category mistake; content has satisfaction conditions, and can be correct or not. Vehicular properties contain structural information [*logons* in MacKay’s (1969) terminology], which is used to control the action and make decisions. Hence, it becomes semantic or control information. As long as actions fail, the reward expectancies generated by place cells are falsified and the animal’s ventral striatum sends appropriate prediction error signals to the actor. This is how the truth value depends asymmetrically on vehicular processes of comparing the predicted reward with the actual reward signal. Referential opacity is possible, which in turn relies on characteristics, and this fulfills the second criterion. Consequently, we have a naturalistically credible story about satisfaction conditions of content.

## Intermediary conclusion: anticipatory mechanisms and the Hard Problem of Content

Anticipatory mechanisms are a subspecies of the general category of representational mechanisms. It is particularly important for biological reasons, valuable for planning ahead and avoiding future danger. In the case of navigation in rats, the account of anticipatory mechanism, along with the empirical evidence cited, vindicates representational ideas about using cognitive maps to predict the near future. Granted, current knowledge of neural mechanisms in rats is incomplete and the overall organization of navigational system in rodents may be different from how it is hypothesized today. But for my purposes this is not particularly important. The point was to show that anticipatory representational mechanisms are explanatorily and predictively relevant entities, and they are parts of our current best theories of rat navigation. By assuming that they operate during navigation, we can make robust predictions. For example, there will be a specific kind of anticipatory spiking or rats can be in error when placed in geometrically similar mazes.

By looking at how place cells help predict the near future by prospectively representing future paths in their environment, and how they evaluate these paths to choose the best route, we can see how an anticipatory mechanism may work in detail, and also in a basic, prelinguistic mind. There is compelling evidence that decision-making and reinforcement learning require forward models to control action. Thus, basic minds without content, touted by REC, such as the “minds” of amoebas or slime mold, are not particularly exciting for cognitive science. Most animals have representational mechanisms with content that has satisfaction conditions—conditions evaluable by reward-related mechanisms in the animal.

The main argument for REC—that correlation or similarity do not constitute content—is cogent but remains irrelevant. Note that correlation and similarity (structural informational relationships in general) are used to discover the structure of vehicles of representation in the current proposal. In an anticipatory mechanism, the truth bearer is neither the structural informational relationship itself, nor the vehicle, but the content of the expectation that some future state of affairs will be such and such. The expectation may have a format of S-representation in Cummins’s (1996) sense, which means it is a model based on structural similarity relationships, and one can decide the format of the representation by inquiring the way the expectation is evaluated in the animal. When place cells are used for navigation in a maze, they generate an expectation about the location of the goal (linked with a reward) and a possible trajectory to the goal. The content of this expectation is non-linguistic and is encoded in spatiotemporal relationships of spikes in the place field. For all we know about the functioning of the hippocampus, if the place field neurons were activated the way they normally do in a certain environment *E*_1_, the rat would behave as if it were in *E*_1_, rather in some other environment *E*_2_. The behavior of the rat is causally related to the spiking of place fields, irrelevant of the fact whether the spiking is causally connected to *E*_1_ or not. So, the rat may be in error. Mere structural informational relationship is, indeed, not enough to constitute content. Only if the vehicles of information control the behavior of the agent and are evaluated as controlling the behavior properly or not, do they have satisfaction conditions. Once control information is evaluated, which can be realized computationally on a vehicular level, it has satisfaction conditions. Obviously, the vehicles must have their structure caused by something, and there might be similarity relationships between vehicles and referents involved. However, it is the evaluated control that constitutes content.

## Possible objections

*The satisfaction conditions are presupposed here rather than explained causally.* Note that the specification of the *explanandum* phenomenon indeed presupposes that there are satisfaction conditions. But this is what *explanandum* is: this is something to be explained, and the task of causal explanations is to explain how satisfaction conditions arise. They do so because there are vehicular parts and computational operations that perform discrepancy checks on representation vehicles. Obviously, one can always deny that such vehicular operations have anything to do with semantic satisfaction conditions, but this would be as silly as denying that proof-theoretic algorithms are truth-constrained. Granted, in sufficiently rich calculi, there are logical truths that cannot be decided using proof-theoretic algorithms, but this does not mean that these algorithms do not justify our claims about all other true logical propositions. They do so because truth values systematically depend on vehicular, syntactical properties of logical notation. Note also that mechanistic explanations of rich representational phenomena involve information processing on multiple levels of organization, and in multiple subcomponents (such as the actor or critic module in the actor–critic architecture).

*There is no job for the representation in the sweeps of the hippocampus.* According to Ramsey (2007), as long as there is S-representation, the job description challenge has been met. Additionally, Cummins and Roth (2012) argued plausibly that cognitive maps are S-representations. But there is a deeper answer; the structure of the place field spikes enables the animal to plan its future behavior. It involves characteristics of the targets. In addition, the content is shown to have satisfaction conditions in a naturalistic, purely causal fashion. Hence, the challenge is met.

*There is no content in forward models.* The falsification relies on simple discrepancy detection, so one might be tempted to view it as syntactic only, and not semantic. But it’s not *only* syntactic, as the reward signals are not merely signals that there was prediction error. They are signals that the action of the animal is appropriate or not; they have special biological significance. One can explain the action of the rat as learning to find liquid chocolate in the maze (a reward) and not as merely transforming sensory signals, generating spikes, and passing signals between the hippocampus and the ventral striatum. The latter activities are typical for the computational part of the representational mechanism, but the explanation of the computational properties of the brain does not explain why the brain is directed towards reward rather than otherwise. In a generic action prediction framework, an animal can easily predict that it will die of thirst without drinking, and as long as the animal is thirsty, there would be no prediction error. This is a version of the dark room problem for the predictive coding (Friston et al. 2012). But real rats actively seek fluids in such a situation because the reward information is related directly to their well-being; it has basic physiological significance. We explain their behavior as adaptive, and in this explanation, the story about the reward is an important complement of the merely informational or computational story about the brain. The computational mechanism is embedded in a living organism that uses it to process information and to represent the consequences of its actions. Satisfaction conditions, naturalized, are linked with reward signals in this case.

*This is non-interesting, low-level stuff.* Rats are much simpler than human beings, and their hippocampus is different. But REC claims that basic minds are without content. The present paper, as well as Shea (2014), demonstrates that all minds capable of reinforcement learning and having prospective functionality of the hippocampus are representational. The data about adult human beings is controversial as they are no longer basic minds in REC sense; human beings are essentially social and linguistic, so the claim that semantic content is merely linguistic couldn’t be defeated by reference to humans. In addition, the debate about content in basic minds shouldn’t involve complex issues of folk psychology and propositional attitudes. For these reasons, basic animal minds are the best starting point. The content in place fields is not *ghostly* and has satisfaction conditions without any need to refer to any social or linguistic practices.

## Conclusion

The present account of representational mechanisms is sufficient to answer Ramsey’s challenge and to solve the Hard Problem of Content. It points out that there are at least two ways in which representations are causally relevant: (1) by being referentially opaque, they are indispensable for explaining behavior, especially in cases of misrepresentation, and (2) when their being in error is detectable by the system, the account shows that the system does treat them as representations. Representational mechanisms are not just useful for predicting and explaining the system’s behavior; they are also specifically representational. Their content has satisfaction conditions.

The idea that neural structures in basic minds might be representational as long as they control behavior and are related to reward mechanisms is not just convenient, and my argument for using it is not based on the assumption that more facts can be subsumed under representational generalizations than under neural generalizations (Pylyshyn 1984). By positing representational mechanisms, we may have a similar level of generality as before. But generalization is not the most important factor for explanation. After all, generalization may occur at the price of abstracting from details, and the account of navigation in terms of simple taxis or tropisms could be extrapolated from invertebrates to rodents, thus yielding a higher level of generalization.

By using the account of representational mechanisms we may discover the function of neural organization and understand why error-correcting pathways exist in the first place. There is no real worry that the notion of representation may be eliminated here; the function of neural mechanism is to represent, and to establish this one need not warrant a gap between representational explanations and neural explanations. In this case, integration of evidence at multiple levels in the organization of mechanisms is much more important than the purported autonomy of individual levels.

Satisfaction conditions are not constituted by structural informational relationships, which are frequently discussed in the debate on content determination, but they are essentially linked with control and evaluation. Both the job description challenge and the Hard Problem of Content are solved by a naturalistic account of satisfaction conditions in representational mechanisms. Although there is much more to be said about satisfaction conditions, and about different possible kinds of representational mechanism—I have provided some detail only for a particular anticipatory mechanism recruiting S-representations—I think the overall story is also plausible. The vindicated notion of representation is not as liberal to cover Watt governors or mouse traps but selective enough to include a large number of basic minds.

## Footnotes

1 MacKay defines structural information content as “number of *distinguishable groups or clusters* in a representation—the number of definably independent respects in which it could vary—its *dimensionality* or *number of degrees of freedom*” (MacKay 1969: 165). By representation, he means anything that has features that “purport to symbolize or correspond in some sense with those of some other structure”. His notion is not to be confused with a stronger notion used throughout this paper.

2 In Fodor’s account, the syntactical properties of vehicles of content are supposed to play the role traditionally assigned to Fregean senses or modes of presentation. This, however, presupposes that homonymy of tokens of Mentalese is impossible. However, neural codes in the brain are highly context-dependent, and one cannot rely on their vehicular properties to be one-to-one mapped to their contents; neither in firing rate encoding, nor in temporal phase encoding; cf. (Rieke et al. 1999). Irrelevant of the question whether homonymy is possible or not, vehicular properties do not have satisfaction conditions, and content has. Solving the Hard Problem of Content requires a substantial account of how satisfaction conditions are possible. I will return to the question of vehicular properties and content at the end of "Anticipatory mechanisms" section.

3 Rosen makes some assumptions that are not exactly essential for the modeling relationship; for example, he doesn’t acknowledge that one could model a natural system in a non-causal manner. The definition of a modeling relationship should not decide whether non-causal modeling is really modeling or not; it simply needs an additional argument, and Rosen fails to offer one. But even opponents of the idea of causality in fundamental physics (Russell 1912; Ross and Spurrett 2007) need not reject Rosen’s analysis of modeling if they simply substitute ‘causation’ with any term they think would best describe the basic structure of physical processes. Also, the notion of inference needs not be understood as entailment in a logical calculus (after all, not all formal systems are logical calculi, and not all computation in formal systems is simply derivation of steps in a proof, even if all formal systems can be described by logical calculi); all we need is that there is an algorithm described by the formal system, which encodes measured values, and decodes them in terms of physical quantities. But note: Non-encoded values, or values that were not measured, are abstracted away, and ignored in models, yet this does not undermine the modeling relationship.

4 Rats, according to some (Naqshbandi and Roberts 2006), lack prospective memory, or the kind of memory involved in planning future action in larger timescales, even if there is some indirect evidence for this (Wilson and Crystal 2012). Whatever the case may be, anticipation in representational mechanisms is not required to span larger timescales.

5 This occurs in so-called theta phase precession; a relatively accessible account of the phenomenon as related to place cells and reward can be found in van der Meer and Redish (2011).

6 At the same time, this difference is mostly terminological; “representation” is understood here as necessarily involving evaluation mechanisms.

7 According to Dayan and Berridge (2014), even Pavlovian learning is model-based. This only strengthens the case for representationalism.
